# Effect of Common Mouthwashes on Mechanical Properties of Suture Materials Used in Dental Surgeries: A Laboratory Experiment

**DOI:** 10.3390/polym14122439

**Published:** 2022-06-16

**Authors:** Shahabe Saquib Abullais, Shaker Saleh AlOsman, Sultan Mansoor AlQahtani, Abdul Ahad Khan, Rakhshinda Nahid, Sulphi Abdul Basheer, Ahmad Saib Jameel

**Affiliations:** 1 Department of Periodontics and Community Dental Sciences, College of Dentistry, King Khalid University, Abha 61421, Saudi Arabia; drsaquib24@gmail.com; 2 Inters, College of Dentistry, King Khalid University, Abha 61421, Saudi Arabia; sheko7727@gmail.com (S.S.A.); s1_m3@hotmail.com (S.M.A.); 3 Department of Oral and Maxillofacial Surgery, College of Dentistry, King Khalid University, Abha 61421, Saudi Arabia; subasheer@kku.edu.sa; 4 Department of Periodontology, Faculty of Dental Sciences, Institute of Medical Sciences, Banaras Hindu University, Varanasi 221005, India; rakhshindanahidbhu@gmail.com; 5 Department of Prosthetic Dental Sciences, College of Dentistry, Jazan University, Jazan 45142, Saudi Arabia; ahmadsaib11@gmail.com

**Keywords:** oral surgery, periodontal surgery, suture, mouthwash, mechanical properties, in vitro

## Abstract

Background: Sutures play a pivotal role in promoting healing in any surgical procedure. However, the versatile nature of the oral environment entails additional properties in the suture materials to assure uneventful healing. A surgeon needs to understand these delicate intricacies in the immediate postoperative phase and needs some kind of algorithm in the selection of sutures that suits the different surgical scenarios. Therefore, a study was designed to observe the effect of four different types of mouthwash, viz. Avohex, Aloedent, Parodontax and Betadine, on the mechanical properties of Vicryl (polyglactin 910), PTFE (polytetrafluoroethylene), Prolene (polypropylene) and Mersilk. Methods: A total of 288 suture samples were divided in four equal groups (72 = each group) and used for the experiments. Tensile load, tensile strength as well as percentage elongation of the suture materials were evaluated in the various stated media at 3rd, 7th, 10th and 14th days, which were compared with the controls. The results were analyzed statistically and inferences were drawn. Results: Mersilk in (Avohex, Parodontax and Betadine) solutions showed a statistically significant decrease in tensile load over time. All the sutures had the highest tensile strength in Parodontax and had the lowest in Betadine solution. Vicryl and PTFE presented significant percentage elongation over time as compared to Prolene and Mersilk. Conclusion: The author has given certain recommendations but also states that there is no standard rule for the use of a particular suture material in all clinical situations. However, the selection of the suture material should be based on the type of surgery and preferences of the surgeon.

## 1. Introduction

Sutures are consistently being used for wound approximation in periodontal and oral surgical procedures for wound closure. In 3500 BC, the use of linen as suture material was first described by the Egyptians. Animal hair, vegetable fibers, silk, leather and gut have been successfully used as suture materials for wound closures [1]. Most sutures are made up of polymers. Polymers are large molecules made up of repeating units of small molecules called monomers. Sutures can be classified in several ways. Based on the raw material used, they can be either natural or synthetic. Depending upon their structure, they can be either monofilament or multifilament. Based on their resorptive and degenerative capacity, they can be categorized into absorbable and non-absorbable. Non-absorbable sutures resist their dissolution in saliva, oral fluids and serum. Moreover, a second appointment is required for their removal. Absorbable sutures are degraded by hydrolysis or by proteolytic enzymes, hence their removal is not essential [2,3].

Vicryl (Polyglactin 910) is a commonly used synthetic absorbable (resorbable) suture material obtained from a copolymer of 90% glycolide and 10% l-lactide. Polytetrafluoroethylene (PTFE) is a biologically inert, synthetic, non-absorbable (non-resorbable) suture material consisting of fluoropolymer of tetrafluoroethylene. Mersilk (silk) is a natural, non-absorbable suture material that is frequently used in oral and periodontal surgeries because of its superior handling characteristics [4,5]. Prolene (Polypropylene) is a synthetic, nonabsorbable suture material that is made from a polymer of propylene [5]. 

After the surgical procedure, the flaps are placed in close approximation with the help of sutures for an intended period of time; this is important to achieve healing through primary intention. Improper positioning of flaps results in healing by the secondary intention, which may compromise the results. The ideal suture material demands several specific characteristics, such as having adequate tensile strength, having the capacity to elongate with tissue edema, being biocompatible, being easy to manipulate while using, able to maintain a secure knot, and should biodegrade if used in internal wound closures [6,7]. Among the various intrinsic properties exhibited by a suture material, the tensile strength and percentage elongation are the most important in order to maintain the surgical flaps in position during the wound healing phase [8].

In oral surgery, suture induced wound healing is difficult as compared to the other parts of the body because of factors related to saliva, quality of the tissue involved, high level of vascularization and the dynamics of speech, mastication and swallowing [9,10]. In the oral cavity, significant variations in pH and temperature are inevitable due to the consumption of various types of foods and fluids. This variability is also caused by the use of prophylactic agents such as toothpastes and mouthwashes. Different suture materials exhibit dissimilar characteristics and soft tissue responses in the oral cavity [3,11,12].

A dental surgeon needs to understand the physical and mechanical properties of commonly used suture materials. At present, no suture fulfills all the requisites of an ideal suture material [13]. Most of the published data on the tensile behavior of sutures mainly focus on the tensile strength. Comprehensive reports on other vital properties of suture material such as percentage elongation, modulus and full stress-strain curves are quite limited in the field of periodontal and oral surgery.

In the present study, one absorbable and three non-absorbable suture materials were studied: Vicryl (polyglactin 910), PTFE (polytetrafluoroethylene), Prolene (polypropylene) and Mersilk (natural silk). The purpose of this study was to compare the effects of four different types of mouthwashes, namely Avohex, Aloedent, Parodontax and Betadine, on the mechanical properties of the above said suture materials, which are commonly used in periodontal, oral and maxillofacial surgeries.

## 2. Materials and Methods

The present in-vitro experimental study used four types of suture materials, out of which three were non-resorbable (silk, polypropylene and PTFE), whereas polyglactin 910 was resorbable (Table 1). All the suture materials used in the study were of uniform gauge (4-0). The type of suture material and their gauge were selected because of their frequent use in periodontal and oral surgery. The experimental design was presented to the institutional Ethical Review Board (King Khalid University, Abha, Saudi Arabia) to procure the ethical approval [SRC/ETH/2017-18/090]. The study was carried out between June 2021 and December 2021.

Four different types of thermostatically controlled test media, composed of mouthwashes, were used to evaluate the physical properties of these suture materials. The above setting was devised to simulate potential short-term intraoral exposure to various tested mouthwashes. The experimental media used were as follows:

Test group 1 (TT1) = Avohex; main ingredient: 0.2% chlorhexidine (Avalon Pharma, Sulimaniyah, Riyadh).

Test group 2 (TT2) = Betadine; main ingredient: 1% Povidone Iodine (Mundi Pharma, Alvarado, Argentina).

Test group 3 (TT3) = Aloedent; main ingredient: Aloe Vera (Optima naturals, Vergiate, Italy).

Test group 4 (TT4) = Parodontax; main ingredient: Aqua, glycerin, other (GlaxoSmithKline, Bradford, UK).

Artificial saliva was formulated by assimilating specific chemicals as mentioned in Table 2 in 1000 mL of distilled water [14]. Artificial saliva was preserved in an amber container until used for the experiment. During the experiment, the prepared artificial saliva was mixed with human serum (Human AB serum, Equitech-Bio, Kerrville, TX, USA) in the ratio of 1:1 concentration at 37 °C and a pH of 7.4 to 8.1 [6] to mimic the biologic simulation of the oral environment. 

**Figure 1:**
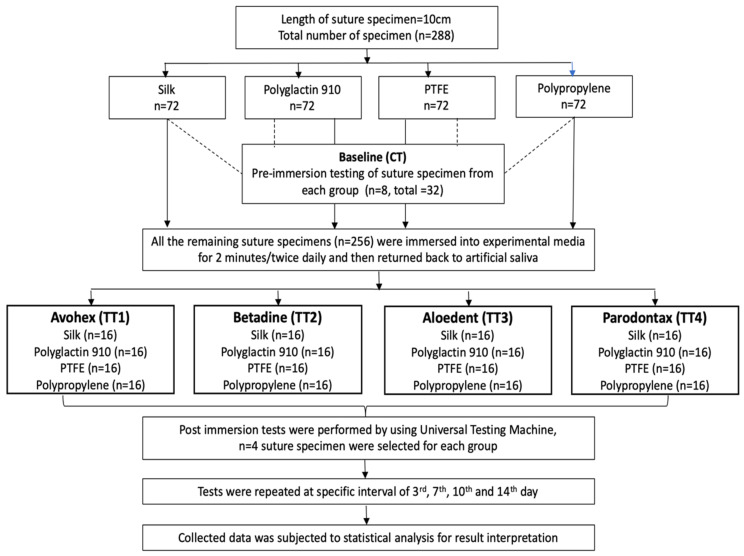
Flow chart of the study.

A total of 288 suture specimens were collected from sterile, unexpired and commercially available individual packs. All the suture specimens were cut to a uniform length of 14 cm. From each group, eight specimens were tested in the dry state for tensile strength (TS) before immersing it into the test media and were labeled as the control group. The rest of the 256 specimens were kept in artificial saliva until they were exposed to the test media. All the suture specimens were exposed to the test media for 2 min twice daily and returned to the artificial saliva after washing with normal saline. The comprehensive study protocol has been explained in Figure 1.

**Figure 2:**
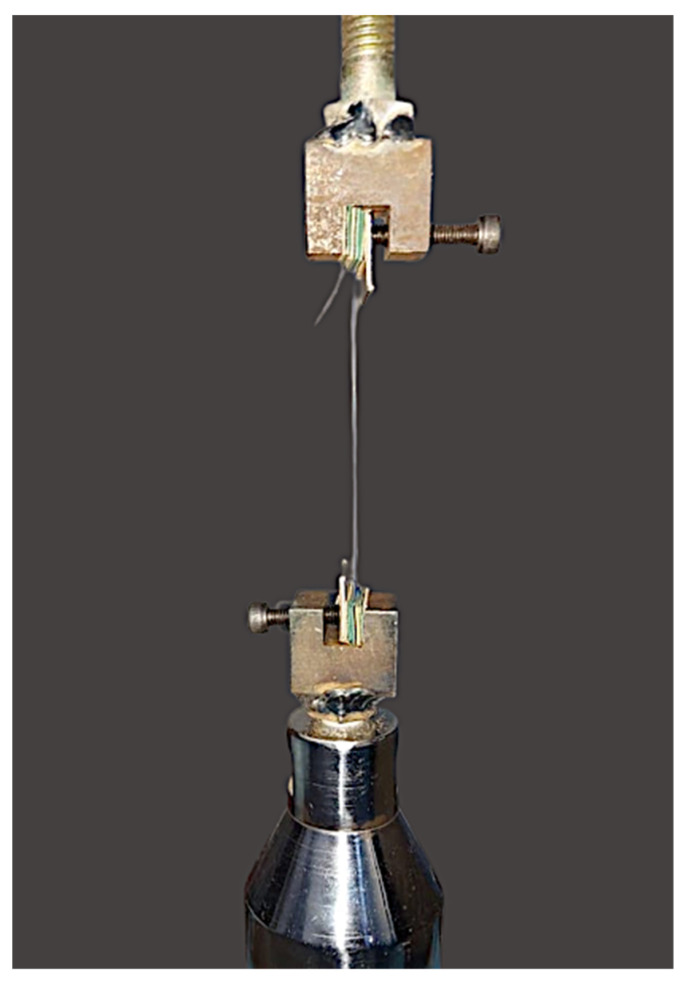
Suture specimen mounted by using modified attachments between the crossheads of universal testing machine.

TS of the suture specimens was tested by the Universal Testing Machine (UTM) (Star Testing Systems, India. Model No. STS-248) which was connected to a computer for digital readout (Figure 2). TS of the suture specimens was assessed at specified time intervals: pre-immersion (dry), followed by post-immersion into test media on the 3rd, 7th, 10th and 14th day. Each suture specimen was secured by a modified jig between the opposite arms of the machine with a fixed distance of 10 cm. Pilot testing revealed that there was no slippage of jig or specimen failure by the above arrangement. TS assessment of each suture specimen was carried out at a cross-head speed of 5 mm/min. Each specimen was stretched until the material broke apart. The maximum load required to break the suture was documented in Newtons (N). The collected data was organized and subjected to statistical analysis.

The data on continuous variables were presented as mean and standard deviation across the 4 study groups. The intergroup statistical comparison of means of continuous variables was conducted using analysis of variance with Bonferroni correction for multiple group comparisons. The intragroup statistical comparisons were conducted by repeated measure analysis of variance in each study group. The underlying normality assumption was tested before subjecting each variable to the analysis of variance and repeated-measure analysis of variance. In the entire study, the p-value of less than 0.05 was considered to be statistically significant. All the hypotheses were formulated using 2-tailed alternatives against each null hypothesis (hypothesis of no difference). All data were statistically analyzed using Statistical Package for Social Sciences (SPSS version 22.0, IBM Corporation, New York, NY, USA) for MS Windows.

## 3. Results

At the end of the soaking period of suture materials in their respective media, all were intact and suitable for mechanical testing. Each suture material represented a unique apparent breaking point, firmness and elongation while being tested.

### 3.1. Tensile Load

All the values of tensile load for different suture materials in different test media are displayed in Table 3. From the table, it can be observed that the values of tensile load are maintained for all suture materials, in different solutions over a period except for Mersilk (in Avohex, Parodontax and Betadine solution) which shows a statistically significant decrease in tensile load over time.

Upon comparing the tensile load for different solutions within the group, it was noticed that PTFE and Mersilk both showed statistically significant differences on the 7th day, while for Prolene it was on the 14th day. All three specimens had the highest value for Parodontax and the lowest for Betadine. Vicryl shows a significant difference on the 10th and 14th day, having the highest load in Parodontax and the lowest in Betadine solution at both periods.

### 3.2. Tensile Strength

The mean values of TS for each suture material in different solutions at different periods are shown in Table 4. From the table, it can be observed that PTFE, Vicryl and Prolene maintained their TS at different periods, and the difference in TS over time is statistically nonsignificant. However, Mersilk shows a statistically significant decrease in TS from baseline until day 14th. 

Upon intragroup comparison of TS in different solutions, it was observed that PTFE showed a statistically significant difference on day 7, Prolene on day 14 and Mersilk on day 7 and 14. All the above sutures had the highest strength in Parodontax and lowest in Betadine solution. Vicryl displayed a statistically significant difference at each period, having the highest value for Parodontax and the lowest for Betadine.

### 3.3. Percentage Elongation

Table 5 shows the values of percentage elongation of different suture specimens in different media over the stated period. From the table, it is found that different suture-material specimens showed a statistically significant difference in percentage elongation in different test media over the stated period. PTFE showed a statistically significant difference in percentage elongation over the period for Aloedent and Betadine solutions, but Vicryl shows a statistically significant difference in Betadine solution only. Meanwhile, Prolene and Mersilk show statistically significant differences in Aloedent and Parodontax solution at different time periods, respectively.

Upon intragroup comparison, PTFE showed a statistically significant difference in percentage elongation on the 7th and 10th day, whereas Vicryl showed a statistically significant difference on the 3rd, 7th and 10th day. However, Prolene and Mersilk showed significant differences on the 3rd and 7th day, respectively.

## 4. Discussion

A suture is a medical tool used to hold body tissues together after an injury or surgery. Most sutures are made of polymers. Polymers are made up of large number of individual repeating units called monomers. If individual repeating units are from one monomer, they are called homopolymers, whereas if they are made from a combination of two or more different monomers then they are called copolymers. The properties of a polymer depend on the structure of the monomer(s) used, the size of the polymer chain, and in the case of a copolymer, the composition and positioning of each repeating monomer chain [15]. The properties of the suture are determined by the molecular structure of the polymer and how the chains of polymers interact with each other. The structural characteristics of the polymer such as molecular mass, degree of entanglement, intermolecular forces and hydrogen bonding can affect the properties such as quality, drawability and strength when used to make a suture [15].

Clot formation and stabilization in the immediate postsurgical phase is an inevitable part of uneventful healing. Sutures play a pivotal role in achieving this objective. The biomechanical properties of the suture materials play a crucial role to neutralize the physiological tensile forces acting on the margins of the healing wound [16,17].

The purpose of this study was to compare the effects of four different mouthwashes, namely Avohex, Aloedent, Parodontax and Betadine, on the mechanical properties of silk, polypropylene, PTFE and Polyglactin suture materials. A single examiner carried out the entire study to minimize variability. The methodology used for this research was established according to previous studies [18,19]. Further, a 1:1 ratio of serum and saliva was used in our study to simulate possible oral surgical and periodontal surgical situations [6,20].

TS of suture material can be defined as the ratio of maximum (tensile) load that a suture can withstand without breaking, while being stretched to the original cross-sectional area of the given material [21]. It can be observed from the results that all suture materials maintained their tensile load in different solutions except Mersilk, in which the tensile load reduced over time. Similarly, it can be observed that PTFE, Vicryl and Prolene maintained their tensile strengths at different time periods, whereas Mersilk shows a statistically significant decrease in tensile strength from baseline until day 14. The reason could be that Mersilk is obtained from natural sources, so maybe more susceptible to the action of proteolytic enzymes from the bacteria as well as the host, whereas others are synthetic. Mersilk is not a polymer and is mainly composed of 70% protein fibers and 30% extraneous material or gum, whereas other suture materials are polymers. Furthermore, Mersilk is braided to increase the knot stability, which is very important during the wound-healing phase. 

Previous studies have reported that absorbable materials have higher resistance to tension while nonabsorbable ones show lower resistance [22]. Khiste et al. inferred that polyglactin (Vicryl) sutures maintained their tensile strength until the 10th day but had minimal strength by the 14th day [23]. Another study by Ferguson et al. reported diminished tensile strength of Vicryl in saliva as compared to immersion in other liquids such as soy, saline or milk [24].

However, Chu et al. report that Vicryl shows better breaking strength as compared to natural sutures. This is especially evident after immersion in physiological and acidic pH solutions. They also reported that from among the nonabsorbable sutures, silk appears to be the most susceptible to various pH conditions [25]. Jose Arce et al. have stated in their research that Teflon (PTFE) and polyglactin (Vicryl) sutures maintained their strength after 21 days of evaluation [26]. On the other hand, the monofilament and Polyglactin (Vicryl) displayed higher tensile strength as compared to BS (black silk) and PTFE [27,28,29].

Chu et al. also reported that pH levels effected the performance outcome of absorbable sutures more than nonabsorbable ones. They further elaborated that both acidic and alkaline environments are able to accelerate the degradation of absorbable sutures [25]. Meanwhile, according to Ven Herdeen, only alkaline conditions accelerated the degradation of synthetic, or “man-made,” absorbable sutures [30].

Kearney CM et al., while evaluating the effect of various equine physiological and pathological fluids, on the rate of degradation of commonly used suture materials, found that multifilament suture materials demonstrate more rapid degradation as compared to monofilament sutures [31].

Percentage elongation or strain of a material describes the change in length of that material, as compared to its original length, before the breaking point is reached [31]. In our study, PTFE shows a statistically significant difference in percentage elongation over time for Aloedent and Betadine solutions, whereas Vicryl shows a difference in Betadine solution only. Meanwhile, Prolene and Mersilk show a difference in Aloedent solution and Parodontax solution, respectively.

Kearney CM et al. stated that incubation time significantly affected the percentage elongation for each suture type in all culture media. Further monofilament sutures displayed the highest yield to failure strain as compared to multifilament sutures, including polyglactin. It is also stated that monofilament suture (polydioxanone) exhibits a high level of elasticity in all media as compared to multifilament sutures (polyglactin) [31]. Naleway et al., while evaluating the mechanical properties of suture materials in general and cutaneous surgery, were of the opinion that increased elongation would be advantageous in situations where a great deal of edema is expected postoperatively [7].

We recommend that in spite of the statistically significant reduction in strength of Mersilk on 10th day, it retains approximately 90% of its strength until the 7th day, so can be recommended in surgical situations in which tissue immobilization of 5–7 days is sufficient to promote healing. At the same time, Parodontax should be prescribed alongside it as a mouthwash during the immediate postsurgical phase. However, if prolonged immobilization of 2 weeks is required, then PTFE should be preferred over Vicryl and Prolene. Aloedent or Parodontax should be prescribed as a mouthwash and Betadine should be avoided along with PTFE during the immediate postoperative phase. Further, Vicryl can be recommended if mild-to-moderate tissue edema is expected.

However, our study has a few limitations that restrict the application of the results in the clinical scenario. As it is an in vitro study, although we have tried to simulate the oral environment in/with regard to temperature and pH, we could not incorporate the microbiological component, which could affect our outcome significantly. Besides the effect of the diverse muscular actions related to the functional components of mastication, deglutition and speech could not be replicated in the experimental setup. Further, there is a scope for additional research as to explore the type of suture materials for use in the specific type of tissue.

## 5. Conclusions

Under the limitation of the present study, it can be concluded that various suture materials exhibit different properties under different circumstances, based on the degree of tissue edema expected, the amount of immobilization required, pH variations expected and the mouthwash recommended for oral hygiene maintenance. The recommendation of a particular suture material in all clinical situations, as a rule of thumb, cannot be justified. Therefore, the selection of the suture material should be tailored to the requirements of the surgery and preferences of the surgeon.

## Figures and Tables

**Table 1:** Details about the suture material used for the experiment.

Suture Material	Composition	Degradation	Manufacturer
**Silk**	Natural braided	Non-absorbable	Mersilk, Ethicon, J&J Pvt. Ltd., Mumbai, India.
**Polyglactin 910**	Synthetic braided	Absorbable	Vicryl, Ethicon, J&J Pvt. Ltd., Mumbai, India.
**Polypropylene**	Synthetic monofilament	Non-absorbable	Prolene, Ethicon, J&J Pvt. Ltd., Germany.
**Polytetrafluoroethylene**	Synthetic monofilament	Non-absorbable	Dental-A, Golnit Ltd., Kiev, Ukraine.

**Table 2:** Composition of artificial saliva.

Chemical Constituents	Concentration (mg/L)
Sodium chloride	125.6
Potassium chloride	963.9
Potassium thiocyanate	189.2
Monopotassium phosphate	654.4
Urea	200.2
Sodium sulfate decahydrate	763.2
Ammonium chloride	178.0
Calcium chloride dihydrate	227.8
Sodium bicarbonate	630.8

**Table 3:** Tensile load (N) for each suture material in different solutions at different points (in time).

Materials		Tensile Load (N) (Mean ± SD)					ANOVA
		Initial	3 Days	7 Days	10 Days	14 Days	*p*-Value
**PTFE**	AVOHEX	8.05 ± 0.6	7.69 ± 0.07	7.59 ± 0.07	7.25 ± 0.92	7.19 ± 0.49	0.482 ^NS^
	ALOEDENT	8.05 ± 0.6	7.40 ± 0.06	7.25 ± 0.06	7.96 ± 0.08	7.20 ± 0.50	0.194 ^NS^
	PARODONTAX	8.05 ± 0.6	9.70 ± 1.66	8.59 ± 1.66	6.70 ± 0.81	6.98 ± 1.24	0.105 ^NS^
	BETADINE	8.05 ± 0.6	7.35 ± 0.13	7.00 ± 0.13	6.65 ± 0.64	6.85 ± 0.57	0.105 ^NS^
**ANOVA**	***p*-Value**	-	0.124 ^NS^	0.008 **	0.332 ^NS^	0.956 ^NS^	
**VICRYL**	AVOHEX	25.51 ± 6.9	23.38 ± 2.40	22.83 ± 2.40	22.00 ± 0.92	18.77 ± 0.73	0.242^NS^
	ALOEDENT	25.51 ± 6.9	24.78 ± 1.31	24.10 ± 1.31	21.23 ± 0.98	20.95 ± 0.32	0.359 ^NS^
	PARODONTAX	25.51 ± 6.9	25.20 ± 1.08	24.37 ± 1.08	22.05 ± 0.73	21.27 ± 1.15	0.426 ^NS^
	BETADINE	25.51 ± 6.9	22.28 ± 1.71	22.00 ± 1.71	19.80 ± 0.57	16.45 ± 1.33	0.064 ^NS^
**ANOVA**	***p*-Value**	-	0.216 ^NS^	0.083 ^NS^	0.032 *	0.001 ***	
**PROLENE**	AVOHEX	15.05 ± 1.5	13.42 ± 0.74	14.53 ± 0.74	13.89 ± 0.68	14.11 ± 0.55	0.353 ^NS^
	ALOEDENT	15.05 ± 1.5	16.16 ± 0.36	15.09 ± 0.36	13.62 ± 1.32	14.17 ± 1.36	0.177 ^NS^
	PARODONTAX	15.05 ± 1.5	14.85 ± 0.86	14.24 ± 0.86	13.74 ± 0.55	14.46 ± 0.75	0.596 ^NS^
	BETADINE	15.05 ± 1.5	14.47 ± 2.20	12.29 ± 2.20	12.53 ± 0.71	12.27 ± 0.45	0.074 ^NS^
**ANOVA**	***p*-Value**	-	0.138 ^NS^	0.062 ^NS^	0.281 ^NS^	0.048 *	
**MERSILK**	AVOHEX	13.81 ± 0.5	11.33 ± 0.94	11.04 ± 0.94	10.42 ± 0.87	10.08 ± 0.46	<0.001 ***
	ALOEDENT	13.81 ± 0.5	10.65 ± 2.20	10.97 ± 2.20	10.63 ± 0.55	10.25 ± 0.65	0.018 *
	PARODONTAX	13.81 ± 0.5	12.38 ± 0.30	12.44 ± 0.30	11.34 ± 0.25	10.66 ± 0.57	<0.001 ***
	BETADINE	13.81 ± 0.5	10.38 ± 0.60	10.08 ± 0.60	10.10 ± 0.28	8.97 ± 0.28	<0.001 ***
**ANOVA**	***p*-Value**	-	0.278 ^NS^	0.001 ***	0.110 ^NS^	0.019 *	

*p*-Value < 0.05 is considered to be statistically significant. * *p*-Value < 0.05, ** *p*-Value < 0.01, *** *p*-Value < 0.001, NS—Statistically nonsignificant.

**Table 4:** Tensile strength (MPa) for each suture material in different solutions at different points (in time).

Materials		Tensile Strength (MPa) (Mean ± SD)					ANOVA
		Initial	3 Days	7 Days	10 Days	14 Days	*p*-Value
**PTFE**	AVOHEX	434.27 ± 55.	430.16 ± 3.15	429.61 ± 3.15	440.34 ± 12.05	435.16 ± 11.76	0.997 ^NS^
	ALOEDENT	434.27 ± 55.	418.53 ± 3.92	410.21 ± 3.92	507.66 ± 2.80	464.63 ± 29.92	0.141 ^NS^
	PARODONTAX	434.27 ± 55.	548.80 ± 94.08	634.16 ± 94.08	576.81 ± 85.81	481.53 ± 30.14	0.077 ^NS^
	BETADINE	434.27 ± 55.	415.76 ± 7.84	397.72 ± 7.84	457.39 ± 20.09	401.11 ± 3.34	0.423 ^NS^
**ANOVA**	***p*-Value**	-	0.121 ^NS^	<0.001 ***	0.115 ^NS^	0.075 ^NS^	
**VICRYL**	AVOHEX	1443.10 ± 391	1315.70 ± 103.69	1246.70 ± 103.69	1204.13 ± 21.07	1213.23 ± 88.60	0.530 ^NS^
	ALOEDENT	1443.10 ± 391	1503.63 ± 36.88	1403.93 ± 36.88	1559.50 ± 136.31	1351.97 ± 44.05	0.722 ^NS^
	PARODONTAX	1443.10 ± 391	1651.90 ± 96.20	1448.63 ± 96.20	1532.27 ± 116.81	1355.60 ± 108.31	0.458 ^NS^
	BETADINE	1443.10 ± 391	1260.17 ± 223.94	1266.13 ± 223.94	1159.70 ± 68.59	960.27 ± 58.02	0.150 ^NS^
**ANOVA**	***p*-Value**	-	0.025 *	0.040 *	0.001 ***	0.001 ***	
**PROLENE**	AVOHEX	851.84 ± 86.64	759.45 ± 41.85	822.32 ± 41.85	794.08 ± 75.98	799.26 ± 31.19	0.490 ^NS^
	ALOEDENT	851.84 ± 86.64	914.66 ± 19.99	853.69 ± 19.99	784.77 ± 21.34	801.94 ± 76.88	0.148 ^NS^
	PARODONTAX	851.84 ± 86.64	833.36 ± 36.91	805.64 ± 36.91	822.97 ± 38.26	818.58 ± 42.34	0.895 ^NS^
	BETADINE	851.84 ± 86.64	818.58 ± 124.74	725.60 ± 124.74	816.51 ± 66.60	699.45 ± 13.49	0.138 ^NS^
**ANOVA**	***p*-Value**	-	0.127 ^NS^	0.121 ^NS^	0.808 ^NS^	0.05 *	
**MERSILK**	AVOHEX	781.61 ± 29.34	641.18 ± 52.68	624.55 ± 52.68	581.97 ± 62.62	567.93 ± 51.49	0.001 ***
	ALOEDENT	781.61 ± 29.34	602.38 ± 124.61	620.95 ± 124.61	639.34 ± 3.20	580.21 ± 36.91	0.019 *
	PARODONTAX	781.61 ± 29.34	700.32 ± 16.93	704.01 ± 16.93	642.52 ± 40.80	602.97 ± 32.06	<0.001 ***
	BETADINE	781.61 ± 29.34	587.60 ± 33.72	571.81 ± 33.72	584.52 ± 76.01	474.33 ± 39.02	<0.001 ***
**ANOVA**	***p*-Value**	-	0.277 ^NS^	0.001 ***	0.379 ^NS^	0.02 *	

*p*-Value < 0.05 is considered to be statistically significant. * *p*-Value < 0.05, ** *p*-Value < 0.01, *** *p*-Value < 0.001, NS—Statistically nonsignificant.

**Table 5:** Percentage elongation for each suture material in different solutions at different points (in time).

Materials		Elongation (%) (Mean ± SD)					ANOVA
		Initial	3 Days	7 Days	10 Days	14 Days	*p*-Value
**PTFE**	AVOHEX	43.33 ± 6.7	27.37 ± 11.67	33.31 ± 11.67	26.23 ± 1.24	32.37 ± 8.49	0.176 ^NS^
	ALOEDENT	43.33 ± 6.7	29.94 ± 2.21	49.44 ± 2.21	13.50 ± 2.97	34.13 ± 2.30	0.009 **
	PARODONTAX	43.33 ± 6.7	27.37 ± 18.38	18.28 ± 18.38	17.60 ± 1.27	31.95 ± 1.84	0.068 ^NS^
	BETADINE	43.33 ± 6.7	21.81 ± 2.39	20.55 ± 2.39	22.90 ± 0.42	25.24 ± 2.64	0.004 **
**ANOVA**	***p*-Value**	-	0.896 ^NS^	0.025 *	0.007 **	0.367 ^NS^	
**VICRYL**	AVOHEX	21.54 ± 8.9	21.21 ± 5.69	22.25 ± 5.69	20.91 ± 1.82	20.95 ± 4.64	0.998 ^NS^
	ALOEDENT	21.54 ± 8.9	29.78 ± 0.70	25.96 ± 0.70	23.96 ± 3.34	24.79 ± 3.99	0.351 ^NS^
	PARODONTAX	21.54 ± 8.9	31.00 ± 2.34	24.87 ± 2.34	25.20 ± 0.64	23.79 ± 1.92	0.174 ^NS^
	BETADINE	21.54 ± 8.9	23.96 ± 5.21	38.62 ± 5.21	28.54 ± 2.60	24.89 ± 2.00	0.014 *
**ANOVA**	***p*-Value**	-	0.05 *	0.000 ***	0.024 *	0.482 ^NS^	
**PROLENE**	AVOHEX	32.16 ± 5.6	30.04 ± 1.75	34.75 ± 1.75	35.25 ± 6.28	28.34 ± 0.39	0.298 ^NS^
	ALOEDENT	32.16 ± 5.6	44.54 ± 3.69	33.58 ± 3.69	31.96 ± 2.13	29.62 ± 4.75	0.011 *
	PARODONTAX	32.16 ± 5.6	28.04 ± 3.06	36.08 ± 3.06	32.37 ± 4.36	31.83 ± 2.78	0.689 ^NS^
	BETADINE	32.16 ± 5.6	33.46 ± 7.78	33.30 ± 7.78	41.37 ± 6.30	29.22 ± 0.96	0.195 ^NS^
**ANOVA**	***p*-Value**	-	0.01 **	0.963 ^NS^	0.165 ^NS^	0.507 ^NS^	
**MERSILK**	AVFAOHEX	15.21 ± 2.2	17.96 ± 2.19	17.83 ± 2.19	13.62 ± 2.68	15.00 ± 1.39	0.104 ^NS^
	ALOEDENT	15.21 ± 2.2	15.58 ± 3.72	17.79 ± 3.72	16.46 ± 0.74	12.58 ± 1.92	0.131 ^NS^
	PARODONTAX	15.21 ± 2.2	21.83 ± 5.14	21.08 ± 5.14	16.41 ± 1.30	12.82 ± 1.66	0.009 **
	BETADINE	15.21 ± 2.2	14.91 ± 2.39	16.86 ± 2.39	15.66 ± 0.91	15.60 ± 2.14	0.759 ^NS^
**ANOVA**	***p*-Value**	-	0.153 NS	0.009 **	0.182 ^NS^	0.172 ^NS^	

*p*-Value < 0.05 is considered to be statistically significant. * *p*-Value < 0.05, ** *p*-Value < 0.01, *** *p*-Value < 0.001, NS—Statistically nonsignificant.

## Data Availability

The data presented in this study are available on reasonable request from the corresponding author. The data are not publicly available due to privacy restrictions.
